# Notes and Comments

**Published:** 1898-09

**Authors:** 


### Notes and Comments.[1]

Empyema of the Antrum in an Infant.—Dr. Alexander Douglas reports, in the *Australasian Medical Gazette*, Sydney, New South Wales, a case of empyema of the maxillary sinus in an infant of only three weeks. He was called to see the child who was reported to be suffering from an inflamed eye. On examination, the face presented an unusual and striking appearance; the right cheek was swollen, the right eyeball protruded, the eyelids were hyperæmic, and the conjunctiva congested. The extreme protrusion of the eye suggested a growth of some nature at the back of the orbit. On looking into the mouth he observed that on the right side the roof was bulging into the cavity of the mouth. The superior maxillary bone was prominent in every direction. Pressure over the cheek caused pus to exude from the right nostril. The diagnosis was "empyema of the antrum."

An opening was made inside the mouth external to the alveolus, and pus flowed freely. Subsequently the cavity was washed out by syringing through the opening in the mouth with boracic lotion, free egress being afforded through the opening into the nose.

The case did well. The threatened bursting of the abscess into the region of the orbit was averted by the free drainage; the dis-

[1] The assistant editor solicits contributions for this department,—new methods, new remedies and formulas, or any short practical note which may prove of value to the practitioner or student. Address, 1338 Walnut Street, Philadelphia.

tortion of the right side of the face gradually disappeared, and the right eye has practically returned to the normal level.

---

Desecrators of the Grave.—*The British Journal of Dental Science* calls the late Dr. Thomas W. Evans the "Pecksniff of the European courts." The only comprehensible thing in its editorial of post-mortuary detraction is that it is characteristic. What there was of resemblance in Dr. Evans to that type of English hypocrisy, Pecksniff, it is hard to conceive. He was certainly an entirely reputable man and dentist, and he reflected honor upon dentistry as a profession. Our specialty has a higher status in every country upon the face of the globe, because Dr. Evans lived and practised. This should have protected his memory from the ghoulish attacks of any dental journal, even though Dr. Evans was a successful man and an American, two almost unpardonable crimes in the minds of many foreigners.—*Dental Practitioner.*

---

Artistic Environment for the Dentist.—In a very interesting and instructive interview in the *Items of Interest*, Dr. J. Leon Williams says of the dentist's surroundings, "A dentist's work is, or should be, largely of an artistic nature. It is the direct product of his mind and hand. He should therefore surround himself, so far as is possible, with an environment which tends to foster and cultivate his sense of the æsthetic, not alone for the powerful direct effect of his surroundings upon his work (this is probably the less important influence), but because the constant effect of his surroundings upon his subconscious mind is like the accumulation of force in a storage battery. And it is from this reserve force, so to say, that the impulses for most of our actions proceed. This is not sentiment, but the newest science.

---

Methods of distinguishing Eucaine from Cocaine.—The excessive solubility of hydrochlorate of cocaine permits its being distinguished from hydrochlorate of eucaine, for eucaine is soluble one part in nine of water, while cocaine is soluble in less than its own weight of water. To detect eucaine that may be fraudulently added to cocaine, because of its costing less, Vulpius states that by dissolving ten grains of the suspected salt in fifty cubic centimetres of water, and then adding two drops of aqua ammonia, if the cocaine is free from eucaine the liquid will remain clear, even though a few crystals may be deposited, whilst if eucaine is present the solution becomes cloudy or milky in appearance.—*L'Odontoligique.*

---

How to make One's Self Unpopular.—In writing upon the subject, Dr. T. B. Welch, in *Dental Brief*, says, "If you would be unpopular make frequent mention of your troubles and pains and misfortunes. Speak of your losses and crosses, how people misrepresent and wrong you, abuse and misuse you, misjudge and misunderstand you. Make everything appear doleful, sombre, and forbidding. Only occasionally speak of your blessings and pleasures, your successes and profits, or of anything bright, cheerful, and amusing. Be sure not to let any sunshine into your nature, and specially sure not to let any out, if by any means it should creep in. Be as cold as winter, as dreary as a fall rain, and as dismal as a cold fog. Don't forget to be unapproachable, curt, and reticent; and by all means let your manners be stiff, blunt, and forbidding."

---

A Questionable Act.—In referring to a pamphlet which has lately been circulated, laudatory of a system of crown- and bridge-work, to which is added the certificates of six teachers in dental schools, who append their official signatures as deans, secretaries, and professors, Dr. Barrett, in the *Dental Practitioner*, asks, "Did the various faculties authorize them to do this? If not, they certainly have committed a kind of forgery, perhaps venial because common, and because committed without any base motive, aside from an unseemly vanity. What right has any member of a faculty with which others are connected to compromise them by virtually appending their names to a certificate which they may not approve? Who gave him authority to certify, as dean or professor, that his college approves and endorses an article that is offered for sale? By what right does he, in the absence of a special approving vote, drag the name of an educational institution through the muck of the patent office? Is this case the teachers actually assume to give the college endorsement to a special method of adopting gold crowns *to the six anterior teeth.* Surely their wits must have been wool-gathering when they did such an unprofessional thing as this, —or did the advertiser append their titles without authority?"

